# Theta-gamma tACS used in cognitive impairment in schizophrenia: First results

**DOI:** 10.1192/j.eurpsy.2026.11758

**Published:** 2026-07-31

**Authors:** N. Biačková, A. Čechová, N.-A. Böhmová, J. Jonáš, M. Klírová

**Affiliations:** 1 National Institute of Mental Health, Klecany; 2 Charles University, 3rd Faculty of Medicine, Prague, Czech Republic

## Abstract

**Introduction:**

Cognitive impairment (CI) is one of the major symptom groups in patients with schizophrenia (SCH), spreading across various domains, such as working memory, executive functions, attention, or memory. Endogenous oscillations (EOs) represent one of the basic physiological mechanisms of cognition. Impairment in particular cognitive domains is associated with changes in brain network dynamics and EOs, and there is evidence of their dysfunction in schizophrenia. Theta band and gamma band reduction and gamma band shift, as well as hyperactivation of resting-state theta phase-gamma amplitude coupling have been found in SCH patients. One of the possible interventions is transcranial alternating current stimulation (tACS), which through various mechanisms interferes with EOs.

**Objectives:**

Theta-gamma tACS has not yet been applied in a clinical population, however, it might potentially improve the cognitive functions of SCH patients.

**Methods:**

A randomized, two-arm sham-controlled double-blind clinical trial is currently underway at the National Institute of Mental Health, Czechia. Patients receive a tACS intervention with 10 sessions, lasting 20min, with the intensity of 2mA, combined theta-gamma 6-80 Hz frequency, with electrodes placed at F3-F4. The protocol includes a clinical assessment (PANSS), cognition testing using the Repeatable Battery for the Assessment of Neuropsychological Status (RBANS) and Trail Making Test (TMT-A/B). Furthermore, resting-state EEG and task-based (using Sternberg memory task) EEG is recorded. Evaluation is carried out pre- and post-intervention, and at 1-month follow-up. Target number of included patients is 20. As of September 2025, 15 patients have completed the protocol. This poster will analyse the pilot results.

**Results:**

Preliminary data analysis was done on the first 8 patients on selected parameters of cognition testing, namely Attention and Total score of RBANS. Due to small overall number of participants, only mean and standard deviation was counted, without further statistical analysis. The data (active vs. sham) are not unblided. The mean score for Attention is (Group1/Group2, baseline:end-of-treatment:follow-up): 44,25/49,5 : 55,75/54,25 : 53,25/53,5. The mean Total score is (Group1/Group2, baseline:end-of-treatment:follow-up): 182,75/205,5 : 203,25/219 : 199,75/210,25. These results are only preliminary, as the study is currently running.

**Conclusions:**

This running study is feasible, the intervention method is safe, no major adverse events were noted. There is a difference in cognitive score change between active and sham group in the preliminary and partially blinded data. It can be assumed, that the proposed tACS intervention influences the cognitive functions of SCH patients, although currently it is not clear in which direction, therefore it is not possible to deduce further interpretations. The poster will analyse unblinded data of the whole group of 20 patients.

**Disclosure of Interest:**

This work was supported by grant No. NW26-04-00068 of Ministry of Health, Czech Republic, ERDF-Project Brain dynamics, No. CZ.02.01.01/00/22_008/0004643, Charles University research program Cooperatio-Neurosciences, and grant No. 118724 of the Charles University Grant Agency.

